# Microbial Keratitis at an Urban Public Hospital: A 10-Year Update

**DOI:** 10.4172/2155-9570.1000498

**Published:** 2015-11-30

**Authors:** David T Truong, Minh-Thuy Bui, Pauras Memon, H Dwight Cavanagh

**Affiliations:** Department of Ophthalmology, UT Southwestern Medical Center, USA

**Keywords:** Keratitis, Microbial keratitis, Antibiotic resistance, Corneal ulcer

## Abstract

**Purpose:**

To review the epidemiology, risk factors, microbiologic spectrum, and treatment of microbial keratitis during a five-year period at an urban public hospital with comparison to similar findings a decade earlier at the same hospital.

**Methods:**

Retrospective chart review in the 5-year interval 2009 through 2014 compared to previously reported cases 2000 through 2004 [Eye & Contact Lens 33(1): 45-49, 2007]. Comparative primary outcome measures included best-corrected visual acuity (BCVA), risk factors, culture and sensitivities, treatment, and complication rates.

**Results:**

318 eyes with microbial keratitis were identified. Contact lens wear, ocular trauma, and ocular surface diseases were the most common risk factors. The culture and recovery rates were 73% and 66% respectively. Gram-positive organisms represented 46%, gram-negative organisms 39%, fungal organisms 15%, and Acanthamoeba <1% of corneal isolates. No common corneal pathogens were resistant to aminoglycosides or vancomycin. 48% of cases were initially treated with fortified antibiotics, 43% with fluoroquinolone monotherapy, and 6% with antifungals. 40% of cases received inpatient treatment. At resolution, average BCVA was 20/82 [logMAR 0.61] with 8% of cases resulting in light perception or worse vision. The perforation rate was 8%. 6% of cases underwent urgent penetrating keratoplasty and 4% of cases underwent urgent enucleation or evisceration. Compared to the prior study, significant differences were: (1) lower culture but higher recovery rates, (2) lower admission rate, (3) more contact lens-related cases of *Pseudomonas* ulcers, (4) lower resistance of coagulase-negative *Staphylococcus* to aminoglycoside antibiotics, (5) improved BCVA at resolution, and (6) lower associated complication rates.

**Conclusion:**

Microbial keratitis remains a clinical challenge in the urban public hospital setting. In the past ten years, epidemiology has shifted towards greater contact lens wear with more Pseudomonal infections. Visual outcomes have not worsened despite a shift away from routine culture and inpatient care to fluoroquinolone monotherapy and outpatient management.

## Introduction

Microbial keratitis is a potentially eye-threatening infection characterized by a corneal epithelial defect and underlying stromal infiltrate. The clinical course of the infection depends upon both prompt initiation of effective therapy and the particular pathogen involved [1,2]. The classic treatment paradigm for microbial keratitis has been comprehensive evaluation of the eye including gram stain and culture of corneal scrapings followed by empiric treatment with broad spectrum antibiotics, usually two fortified preparations. Treatment can then be appropriately modified when the causative organism(s) are identified and the antibiotic sensitivities are determined [3].

The availability of highly effective topical ophthalmic fluoroquinolone therapy in the 1990’s has shifted the preferred treatment strategy by most ophthalmologists. Many ophthalmologists no longer culture corneal ulcers on presentation and begin fluoroquinolone monotherapy even when fortified antibiotics are available through local compounding pharmacies [4,5]. Many studies support the therapeutic equivalence or superiority of fluoroquinolone therapy to fortified antibiotics, which can reduce bacterial load by 99.9% within 24 hours [6-9].

While the clinical utility of gram stain and cultures has been limited primarily to the identification of the organism rather than antibiotic sensitivity [10], the emergence of antibiotic resistant microbial pathogens remains a grave clinical concern [11]. Fluoroquinolone resistance has been associated with delayed treatment response [12]. Moxifloxacin and vancomycin have been shown to have equivalent *in vitro* efficacy against methicillin- resistant *Staphylococcus aureus* (MRSA) resistant to ciprofloxacin, but corneal pathogens are now known to harbor moxifloxacin resistance [13].

Periodic surveillance of local treatment and resistance patterns is vital for combating antibiotic resistance and improving patient visual outcomes [1,2,14,15]. We previously reviewed the epidemiology, risk factors, microbiologic spectrum, and outcomes of microbial keratitis at an urban public hospital in North Texas over the 5-year period January 2000-December 2004 [16]. This study reviews current microbial keratitis treatment and provides an update one decade later on the microbiology of infectious keratitis and visual outcomes at that same hospital.

## Materials and Methods

This study was approved by the University of Texas Southwestern (UTSW) Medical Center Institutional Review Board. Medical records of patients treated at Parkland Health and Hospital Systems in the 5-year period between September 2009 and August 2014 with ICD-9 codes for corneal ulcers (370.00, 370.01, 370.02, 370.03, 370.04, 370.05, 370.06, 370.0) were identified and the patients’ clinical history, past medical history, and ophthalmic medical notes were reviewed. Inclusion criteria were patients who underwent a comprehensive ophthalmologic examination and were diagnosed and treated for microbial keratitis. Patients were excluded from the study if they were at any time known or thought to have had a viral or noninfectious keratitis. Data transformations and statistical analyses using Pearson’s correlation coefficient were performed in SAS Enterprise Guide 6.1 (Cary, NC).

Best-corrected visual acuity, spectacle, contact lens, or pinhole, was recorded at both presentation and resolution of the keratitis. Average BCVA was determined by first converting the visual acuity to log of the minimum angle of resolution (logMAR) then taking the average of the logMAR values. Counting fingers vision was converted to snellen equivalent by assuming fingers are the size of a 200 letter. Hand motions vision was considered 10 times worse than count fingers [17]. The patient’s keratitis was considered resolved following urgent penetrating keratoplasty, enucleation, or evisceration, and the vision was not used for calculating average BCVA. Light perception or worse vision was recorded but excluded from average BCVA calculations.

Corneal cultures were performed as previously described [16]. Briefly, calcium alginate swabs were used to directly inoculate specimens onto chocolate agar, blood agar, thioglycollate broth, and Sabouraud’s dextrose agar. Confocal microscopy was performed when Acanthamoeba was suspected. *In vitro* antibiotic susceptibility was performed using the Dade Behring MicroScan system (Deerfield, IL) and interpreted by the serum standards set forth by the Clinical and Laboratory Standards Institute. This information was obtained from the hospital electronic medical record system.

## Results

### Demographics and risk factors: 2009-2014

**Figure 1:**
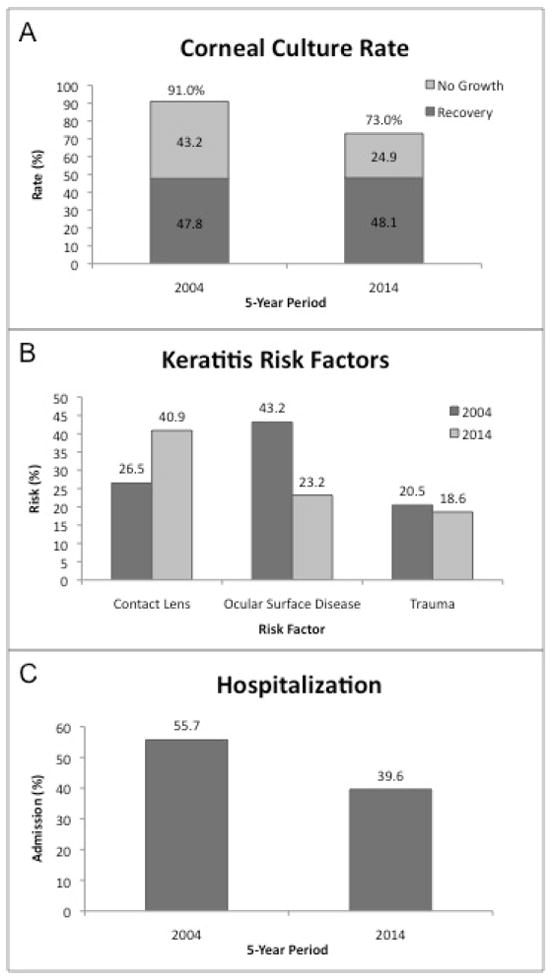
Demographics and risk factors for microbial keratitis. Comparison of 5-year periods, 2000-2004 (n=122), and 2009-2014 (n=318). (A) Culture rate and effective culture positivity rate, (B) risk factors for microbial keratitis, and (C) rate of hospitalization.

Three hundred eighteen patients met the inclusion and exclusion criteria. One hundred ninety patients were male and 128 patients were female. The average age was 42.9 years of age (range, 14-95 years). Two hundred thirty-two patients (73%) underwent corneal cultures and 153 (66%) cultures had positive culture results. One hundred thirty patients (41%) had a history of contact lens wear, 88 patients (28%) had a pre-existing history of ocular surface disease, 55 patients (17%) had preceding ocular trauma, and 13 patients (4%) were on topical steroids in association with development of their corneal infection. One hundred twenty-six (40%) of patients were hospitalized for inpatient treatment. Compared to the prior study, these results demonstrated a lower culture rate but a greater proportion of cultures were positive for causative microorganisms. Contact lens wear was a much greater risk factor for corneal ulcer in this cohort while ocular surface disease was a lesser factor. Fewer patients were hospitalized for their corneal infection (Figure 1).

### Microbiology and antibiotic susceptibility

Corneal culture and confocal microscopy identified 220 organisms. Of the organisms isolated, 46.4% were gram-positive bacteria, 38.6% were gram-negative bacteria, and 14.5% were fungal organisms. One case of *Acanthamoeba* was identified with confocal microscopy. Coagulase-negative *Staphylococcus* was the most common gram-positive bacteria isolated, *Pseudomonas aeruginosa* was the most common gram-negative bacteria isolated, and *Fusariam* was the most common fungal species isolated. Only 3 cases of methicillin-resistant *Staphylococcus aureus* were isolated in culture (Table 1).

**Figure 2:**
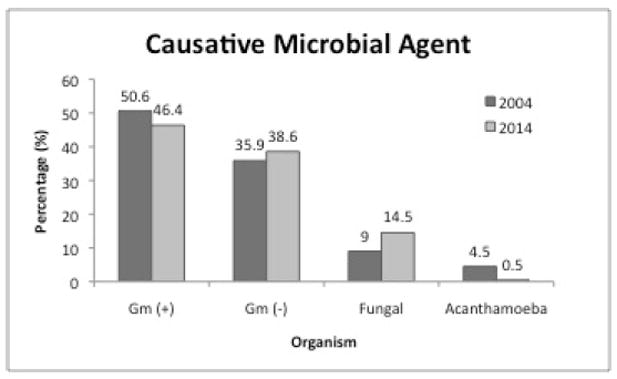
Trends in microbiology of keratitis. Comparison of 5-year periods, 2000-2004 (n=122), and 2009-2014 (n=318), microbiology of microbial keratitis at Parkland Health and Hospital Systems.

Compared to the prior study, there were fewer gram-positive organisms, more gram-negative organisms, more fungal organisms, and much fewer *Acanthamoeba* (Figure 2). *Pseudomonas* infection was strongly associated with contact lens wear (p<0.01). Gram-positive bacterial infection was associated with ocular surface disease and steroid use (p<0.01 and p=0.01 respectively).

Of the bacteria tested, 48.6% were considered to be resistant to erythromycin, 3.8% to ciprofloxacin, 2.3% to gentamicin, and 1.9% to tobramycin. None were considered to be resistant to cefazolin or vancomycin. Of the most common corneal pathogens, only one colony was considered to have resistance to gentamicin. No isolates of *Pseudomonas* were considered to have resistance to ciprofloxacin or levofloxacin. There was widespread resistance to erythromycin among the most common corneal pathogens (Table 2).

### Treatment

One hundred thirty-seven patients received fluoroquinolone monotherapy as the initial therapy for their keratitis (43%); and one hundred fifty-three patients received a combination of fortified vancomycin and gentamicin as initial therapy (48%). Nineteen patients were begun on initial antifungal agents on presentation (6%). One patient was initially treated with polyhexamethylene biguanide (PHMB).

Treatment with fortified antibiotics or prolonged antifungal therapy was associated with inpatient treatment (p<0.01 and p=0.01 respectively) while treatment with fluoroquinolone monotherapy was associated with outpatient treatment (p<0.01). Combination fortified antibacterial therapy was associated with *Pseudomonas* infection (p<0.01). Initial treatment with an antifungal antibiotic was associated with fungal culture positivity (p<0.01) and negatively associated with bacterial culture positivity (p<0.01).

### Outcomes

In the 2009-2014 cohort, BCVA was logMAR 1.43 (20/542) at presentation and logMAR 0.61 (20/82) at resolution of the keratitis. At presentation, 34 patients had light perception or worse vision (10.7%) and at resolution, 26 patients had that vision (8.2%). Twenty-four patients developed corneal perforation (7.5%). One patient died from causes unrelated to their corneal infection. Nineteen patients underwent urgent keratoplasty (6.0%) and 11 patients underwent enucleation or evisceration (3.5%). Fungal and gram-positive bacterial infections, especially with *S. aureus*, were at increased risk for corneal perforation. Fungal infections were at greater risk for urgent keratoplasty than bacterial infections. Gram-positive bacterial infections were at greater risk for urgent enucleation and evisceration than gram-negative bacteria (Table 3).

**Figure 3:**
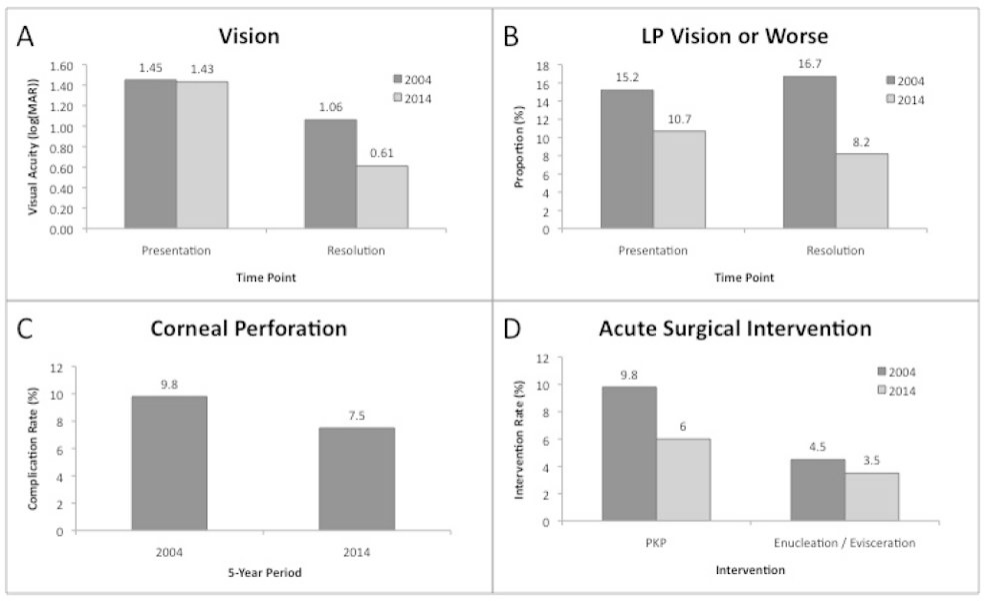
Outcomes of microbial keratitis. Comparison of 5-year periods, 2000-2004 (n=122), and 2009-2014 (n=318). (A) Average best-corrected visual acuity, (B) Proportion of patients with light perception or worse vision, (C) Corneal perforation rate, (D) Rate of acute surgical intervention.

Compared to the prior decade, best-corrected visual acuity on presentation was similar while vision on resolution of the infection was significantly better. The proportion of patients with light perception or worse vision on both presentation and resolution were lower. The corneal perforation rate and rates for both urgent keratoplasty and enucleation or evisceration were lower compared to the prior decade (Figure 3).

## Discussion

### Demographics and risk factors

Compared to the prior decade, substantially fewer patients underwent corneal cultures on initial outpatient presentation. Whereas in the past patients were routinely cultured except in cases of a small or non-vision-threatening infiltrate, currently cultures are often reserved for severe infections, particularly in the community [5]. At our institution, this treatment strategy has resulted in a higher positive culture rate. Of note, similar proportions of the total patients at each study period had a positive corneal culture suggesting the lower risk patients were less likely to have a positive corneal culture.

Ocular surface disease was the most common risk factor for microbial keratitis in the prior decade while contact lens wear is now more common [18]. Not surprisingly, our rates are now more similar to those reported more recently by others [19]. There are now more than 40 million contact lens wearers in the United States and contact lens wear is reported to be the single largest risk factor for microbial keratitis [20,21].

### Microbiology and antibiotic susceptibility

Over the 10-year period, we found an increase in coagulase-negative *Staphylococcus* and *Pseudomonas* and a decrease in *Staphylococcus aureus*, both methicillin sensitive and resistant variants. This pattern has also been found in other studies in the literature [14]. At our institution, we believe this to be due to a greater proportion of patients presenting with history of contact lens wear due to increasing numbers of lens wearers. Despite the increasing alarm over MRSA, there were only 3 cases of MRSA at our institution as compared to 5 cases during the same interval a decade prior; and there has been only 1 confirmed case of Acanthamoeba over the past 5 years. While some studies have found low resistance of coagulase-negative *Staphylococcus* to fluoroquinolones [19], resistance at our institution became so widespread in the past that we no longer routinely test for fluoroquinolone susceptibility. These findings have also been reported by others in the literature [14,22].

While epidemiologically useful, large studies have not shown a relation between antibiotic resistance and clinical outcomes [23,24]. This is not surprising as ophthalmic antibiotic preparations are applied topically at concentrations as high as three orders of magnitude greater than typical minimum inhibitory concentrations with resulting high corneal concentrations.

### Treatment

With concern, routine practice at our institution has also been shifting towards careful monitoring, more selective culturing of patients presenting with microbial keratitis, and initial outpatient therapy with a fourth generation fluoroquinolone antibiotic with close follow-up. This strategy is limited by patient-specific factors in our practice setting but nevertheless, when selectively applied, has not resulted in worsening clinical outcomes. Not surprisingly, we found a negative correlation between fluoroquinolone monotherapy and hospitalization and a positive correlation between fortified antibacterial therapy and hospitalization.

### Outcomes

The average BCVA on presentation did not appear to be significantly different over the 10-year period but the average BCVA at resolution was significantly improved in the latter period compared to the prior period. At the same time, the proportion of patients with light perception or worse vision was lower at presentation suggesting earlier presentation for treatment in the latter population. The proportion of patients with corneal perforation and those requiring urgent keratoplasty or enucleation or evisceration was also lower in the contemporary period.

## Conclusion

Microbial keratitis remains a challenging infection to treat in the urban public hospital patient population despite the availability of highly effective ophthalmic antibiotics. Outpatient compliance is often poor due to a myriad of reasons including poor social support, unstable living situations, medication costs, and inconsistent follow-up. Over the past 10 years, we have found an increasing trend towards a greater proportion of patients with keratitis associated with contact lens wear and with it, a higher proportion of patients with *Pseudomonas*-associated keratitis. Despite widespread clinical concern, increasing numbers of resistant organisms has not been observed at our institution. The MRSA rate and Acanthamoeba rate are unchanged. Treatment patterns have liberalized with less hospitalization and more selective use of corneal cultures. As a result, visual outcomes have not worsened overall but have actually improved.

## Figures and Tables

**Table 1:** Microbiological spectrum of microbial keratitis: 2009-2014. Corneal cultures of microbial keratitis at Parkland Health and Hospital Systems, 5-year period, 2009-2014.

	No.	Percentage (%) of Class	Percentage (%) of Total
**Gram-Positive Organisms**			
Coagulase-negative staph	36	35.3%	16.4%
Non speciated	26	25.5%	11.8%
*Staphylococcus epidermidis*	9	8.8%	4.1%
*Staphylococcus warneri*	1	1.0%	0.5%
Alpha hemolytic *streptococcus*	23	22.5%	10.5%
Non-speciated	10	9.8%	4.5%
*Streptococcus pneumoniae*	13	12.7%	5.9%
*Staphylococcus aureus*	18	17.6%	8.2%
MSSA	15	14.7%	6.8%
MRSA	3	2.9%	1.4%
Group B *Streptococcus*	1	1.0%	0.5%
*Bacillus* species not cerus	5	4.9%	2.3%
*Corynebacterium*	4	3.9%	1.8%
Other gram-positve	15	14.7%	6.8%
Total gram positive	102	100.0%	46.4%
**Gram-Negative Organisms**			
*Pseudomonas aeruginosa*	35	41.2%	15.9%
*Moraxella catarrhalis*	8	9.4%	3.6%
*Serratia marcescens*	6	7.1%	2.7%
*Klebsiella pneumoniae*	5	5.9%	2.3%
*Moraxella lacunata*	5	5.9%	2.3%
*Proteus mirabilis*	3	3.5%	1.4%
*Haemophilus influenzae*	2	2.4%	0.9%
*Neisseria gonorrhoea*	1	1.2%	0.5%
Other gram-negative	20	23.5%	9.1%
Total gram negative	85	100.0%	38.6%
Fungal Organisms			
*Fusariam* species	9	28.1%	4.1%
*Bipolaris* species	8	25.0%	3.6%
*Candida* species	5	15.6%	2.3%
Aspergillus species	4	12.5%	1.8%
Other fungal	6	18.8%	2.7%
Total fungal	32	100.0%	14.5%
Parasitic Organisms			
*Acanthamoeba*	1	100.0%	0.5%
Total Organisms	220		

(MRSA: methicillin-resistant *Staphylococcus aureus;* MSSA: methicillin-sensitive *Staphylococcus aureus*).

**Table 2:** Antibiotic susceptibility of most common corneal pathogens: 2009-2014. Antibiotic susceptibilities of the most common corneal pathogens against commonly available ophthalmic antibiotics. (A) Aminoglycoside antibiotics, (B) Fluoroquinolone antibiotics, (C) Macrolide and cephalosporin antibiotics.

A						

	Gentamicin			Tobramycin		

	Susceptible	Total	Percentage (%)	Susceptible	Total	Percentage (%)

Coagulase Negative *Staphylococcus*	15	15	100.0%	0	0	n/a

*S. aureus*	15	15	100.0%	0	0	n/a

MSSA	12	12	100.0%	0	0	n/a

MRSA	3	3	100.0%	0	0	n/a

*P. aeruginosa*	32	33	97.0%	33	33	100.0%

Resistant Cases	1 case *E. coli*			1 case *E. coli*		

	1 case *P. aeruginosa*					

**B**						

	**Ciprofloxacin**			**Levofloxacin**		

	Susceptible	Total	Percentage (%)	Susceptible	Total	Percentage (%)

Coagulase Negative *Staphylococcus*	0	0	n/a	0	0	n/a

*S. aureus*	0	0	n/a	0	0	n/a

MSSA	0	0	n/a	0	0	n/a

MRSA	0	0	n/a	0	0	n/a

*P. aeruginosa*	33	33	100.0%	2	2	100.0%

Resistant Cases	1 case *E. coli*					
	1 case *Nocardia abscessus*					

**C**						

	**Erythromycin**			**Cefazolin**		

	Susceptible	Total	Percentage (%)	Susceptible	Total	Percentage (%)

Coagulase Negative *Staphylococcus*	9	19	47.4%	9	9	100.0%

*S. aureus*	10	17	58.8%	14	14	100.0%

MSSA	10	14	71.4%	14	14	100.0%

MRSA	0	3	0.0%	0	0	n/a

*P. aeruginosa*	0	0	n/a	0	0	n/a

**Table 3:** Risks for complications and interventions: 2009-2014. Analysis of risk factors and causative organisms for (A) Corneal perforation and (B) Acute surgical intervention.

	Odds Ratio	Confidence Interval (95%)	
N=318		Lower	Upper
**Risk Factors**			
Steroid Use	2.34	0.49	11.24
Trauma	1.84	0.73	4.66
Ocular Surface Disease	1.02	0.39	2.66
Contact Lens Wear	0.18	0.05	0.62
**Causative Organism**			
Fungal	3.16	1.18	8.48
*S. aureus*	2.80	1.09	7.15
Gram (+)	2.43	1.06	5.59
Bacterial	1.91	0.89	4.11
*P. aeruginosa*	0.71	0.16	3.16

		**Keratoplasty**			**Enucleation/Evisceration**		
		**Odds Ratio**	**Confidence Interval (95%)**		**Odds Ratio**	**Confidence Interval (95%)**	
N = 318			Lower	Upper		Lower	Upper
**Risk Factors**							
	Steroid Use	2.94	0.60	14.34	--	--	--
	Ocular Surface Disease	1.87	0.71	4.92	1.79	0.51	6.29
	Trauma	1.14	0.37	3.58	0.94	0.20	4.48
	Contact Lens Wear	0.48	0.17	1.37	--	--	--
**Causative Organism**							
	Fungal	3.82	1.30	11.26	0.75	0.10	5.87
	*P. aeruginosa*	1.88	0.51	6.88	0.59	0.07	4.61
	*S. aureus*	1.42	0.39	5.14	3.53	1.14	10.86
	Bacterial	1.20	0.50	2.85	4.54	1.27	16.22
	Gram (+)	1.07	0.37	3.14	3.52	1.24	10.02
